# Welcome to Materials – a New Open Access Journal for a Growing Scientific Community

**DOI:** 10.3390/ma1010001

**Published:** 2008-08-29

**Authors:** Andreas Taubert

Materials science is one of the key disciplines in modern society because only materials-oriented scientists can provide the necessary tools, building blocks, components, and processes that are needed for an ever growing and aging society. Moreover, supplying the world with energy has become a major issue both for materials scientists and engineers (as well as for politicians). 

Materials research is, by its very nature, a highly interdisciplinary field, where chemists, physicists, biologists, engineers, and others meet to explore the structure, properties, and applications from the single atom or molecule all the way to complex structures such as nano- and microscale devices. Ultimately, at the larger end of the spectrum, no plane will fly without the work of materials scientists. 

The wealth of questions that arises when dealing with problems in the materials field has required the development of sophisticated new equipment and procedures like electron tomography, a technique our colleagues would have loved to have in the 50s to study, for example, defects or grain boundaries. Similary, NMR spectroscopy seems to have evolved in gigantic leaps over the last years. Nowadays, NMR spectroscopy provides a virtually unlimited amount of highly specific information on a material of interest. 

Similarly, new synthetic methods, including genetic engineering and other biotechnological approaches, now allow for the synthesis of molecules and materials that have not been accessible before. Ionic liquids have been one of the key players in the advanced materials field, superconductors keep attracting attention, and carbon nanomaterials have generated tremendous attention. They have also triggered a wave of startup companies focusing on different aspects of carbon nanomaterials around the world. 

Around the world, more and more researchers are engaged in materials research, from simulation to synthesis, from metals to soft matter and the medical field. As the field is ever broadening, more and more specialized journals have appeared. However, in order to enable fruitful interactions between very different fields, there is a need for journals and conferences that cover all aspects of materials research. Furthermore, with a rapidly growing research community all over the world, including countries that have not historically been very active in this area, there is a need to provide easy and cheap access to high quality research for as many scientists as possible.

While in biology and physics, there is already a certain “tradition” of Open Access publishing, chemistry has been rather slow in picking up the concept. This is interesting, because Open Access does have several advantages: free access for anyone interested, color figures can easily be included at no cost (because the journals are published on the web anyway), publication is rapid, and, as the journals can be read by many scientists, including those that do not have access to the expensive subscriber journals, the long term impact is expected to be high.

As a result, the new Open Access journal *Materials* should be of interest to anyone working in the general area of materials synthesis, characterization, and application. I therefore invite you to submit your original, high quality research to *Materials* and hope that with time, *Materials* becomes a premier publication for interdisciplinary materials research worldwide. To achieve this goal, we will work hard and try to provide you with the best service we can. But we also depend on you, the authors, to submit high quality original research that attracts attention. 

